# A Rare Cause of Diffuse Parenchymal Lung Disease together with Granulomatous Reaction: Pulmonary Amyloidosis

**DOI:** 10.1155/2013/837190

**Published:** 2013-02-28

**Authors:** Zuhal Ozer Simsek, Fatma Sema Oymak, Nuri Tutar, Ozlem Canoz, Ramazan Demir

**Affiliations:** ^1^Department of Chest Diseases, Faculty of Medicine, Erciyes University, 38039 Kayseri, Turkey; ^2^Department of Pathology, Faculty of Medicine, Erciyes University, 38039 Kayseri, Turkey

## Abstract

Amyloidosis is a heterogeneous group of disorder associated with the deposition of protein in an abnormal fibrillar form. Primary Sjögren's syndrome (PSS) is a systemic inflammatory disorder that commonly affects the exocrine glands. The reported frequency of pulmonary involvement in PSS varies widely, ranging from 9% to 75%. Pulmonary involvement occurs in light-chain (AL) amyloidosis and is uncommon in the reactive (AA) and hereditary forms. Herein we present a case of PSS associated diffuse multinodular amyloidosis in the lung. We followed up the patient without treatment for three years. There are only minimal lung symptoms related to lung infiltration. In conclusion, pulmonary involvement in SS is an extremely rare clinical manifestation and usually has a good survival rate without treatment.

## 1. Introduction

Amyloidosis is a heterogeneous group of disorders associated with the deposition of protein in an abnormal fibrillar form. The classification of amyloidosis is based on the fibril type [1]. Aggregation of these pathological proteins forms amyloid deposits in various organs eventually leading to organ failure and death. Over 20 amyloidogenic precursor proteins have been documented to form amyloid deposits systemically or localise to specific organs [2]. All amyloid fibrils appear as faintly red on Congo red staining on microscopic examination and show a typical apple-green birefringence under polarised light. The site of deposition relies on the type of amyloidosis, which can be acquired or hereditary, localized, or systemic. At least 25 different precursors of amyloid fibrils are now known [3]. Pulmonary involvement occurs in light-chain (AL) amyloidosis and is uncommon in the reactive (AA) and hereditary forms. The spectrum of pulmonary amyloidosis includes laryngeal, tracheobronchial, parenchymal (localised and diffuse), and mediastinal lymph node disease. Primary Sjögren's syndrome (PSS) is a systemic inflammatory disorder that commonly affects the exocrine glands [4]. The reported frequency of pulmonary involvement in PSS varies widely, ranging from 9% to 75% depending on the detection method employed, and consists of various forms of small airway and interstitial lung diseases [5]. The clinical lung manifestations of PSS include pleuritis, interstitial pneumonia, and fibrosis. The common patterns of lung involvement are honeycomb formation, ground-glass attenuation, centrilobular nodules, reticular pattern, and bronchiectasis [6]. We presented a case with PSS associated diffuse multinodular amyloidosis in the lung. 

## 2. Case

**Figure 1:**
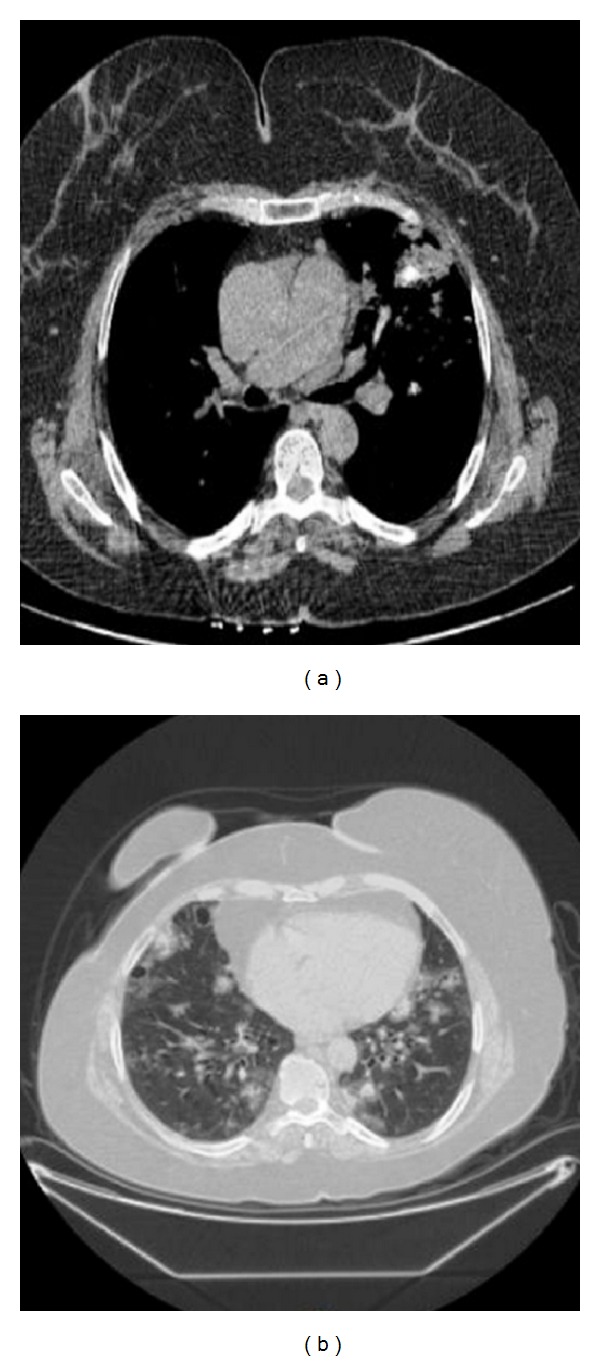
Multiple calcified nodules, air cysts in both lungs, and lymphadenopathy in the mediastinum on chest tomography.

**Figure 2:**
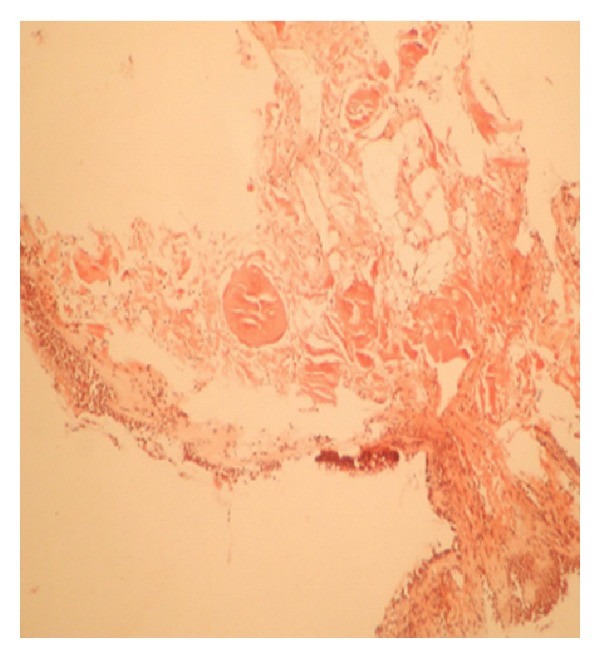
Transbronchial biopsy stained positive with Congo red.

**Figure 3:**
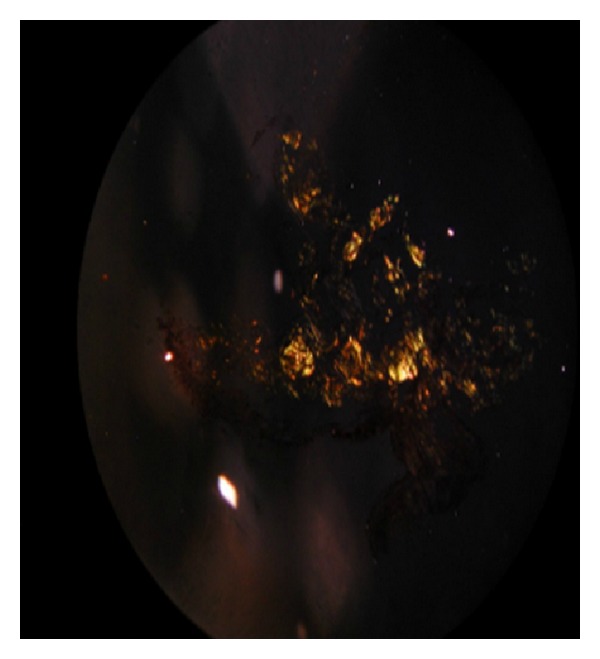
Transbronchial biopsy green birefringence under polarized light.

A 61-year-old woman presented with cough, dyspnea, and hemoptysis of 2-month duration. On physical examination she had macroglossia, multiple subcutaneous skin nodules, clubbing, and on lung auscultation rales in both lungs. She had been diagnosed with dry eye and mouth, positive Schirmer's test, and focal lymphocytic sialoadenitis in minör salivary gland as PSS 10 years previously. The chest tomography showed multiple calcified nodules, air cysts in both lungs, and lymphadenopathy in the mediastinum (Figures 1(a) and 1(b)). Pulmonary function test revealed moderate restrictive ventilatory defect. We performed fiber optic bronchoscopy that showed external compression. BAL fluid specimens were negative for routine bacterial, mycobacterial, fungus, and nocardia. Pathologic evaluation of the bronchoscopy biopsy divulged granulomatous reactions and an amyloid stain (Congo red) (Figure 2) displayed the apple-green birefringence typical of amyloid deposit under polarised light (Figure 3). Hypergammaglobulinemia was observed in protein electrophoresis. Serum immunofixation identified monoclonal immunoglobulin (Ig) A. On bone marrow biopsy, plasma cells made up 10–15% of nucleated cells. We diagnosed PSS related primary systemic amyloidosis (AL). Amyloid infiltration was shown in lung, bone marrow, and skin specimens. There was no proteinuria. We suggested treatment for systemic amyloidosis first high dose steroid follow up oral or parenteral cyclophosphamide but the patient did not agree. We followed the patient without treatment for three years. We performed pulmonary function test each year which was stable during the three years. There are only minimal lung symptoms related to lung infiltration.

## 3. Discussion

Sjögren's syndrome is an inflammatory disorder targeting the exocrine glands and is often accompanied by various systemic manifestations including lung involvement [7]. Pulmonary involvement in SS is being increasingly recognised and may occur in 9–70% of affected patients [8]. About one-third of asymptomatic patients show abnormalities on CT, most frequently bronchiolitis [9]. The etiopathogenesis of lung involvement in PSS patients is largely unknown. There may be more than one cause, including genetic association, alteration of the immunological response, aberrant tissue repair, and environmental factors. Yazisiz et al. [10] have been reported that PSS is more common in females; however, the presence of lung involvement has been seen in the male population and smokers at a high percentage compared to females and nonsmokers. This association could be related to the high frequency of smoking history in male patients. In symptomatic patients, interstitial lung disease (ILD) dominates. Lung involvement is usually a late manifestation and only 5% of patients present with it [11]. Our case presented with lung involvement 10 years after being diagnosed with PSS; it was observed together with multiple calcified nodules, air cysts in the both lungs, and lymphadenopathy in mediastinum on CT. 

Amyloidosis is a heterogeneous group of disorders associated with the deposition of proteins in an abnormal *β*-sheet fibrillar form. The nature of the deposited fibrils has been used to classify the disorders, and 25 different proteins have been recognised to date. Amyloid deposits are identified on the basis of their apple-green birefringence under a polarised light microscope after staining with Congo red. The two most common forms of amyloidosis are light-chain (AL) amyloidosis and reactive amyloidosis (AA) due to chronic inflammatory disease. AA is an acute-phase protein produced in response to inflammation. AL amyloidosis is a clonal plasma cell proliferative disorder in which fibrils are deposited in the kidney and other tissues. In AL amyloidosis, clonal plasma cells in the bone marrow produce light chains that are amyloidogenic. Bone marrow biopsy showed a clonal plasma cell proliferation in presented patient. Nodular parenchymal amyloid deposits usually appear in multiple sites; focal deposits do occur, albeit much less commonly. The patients are generally asymptomatic. Amyloid nodules are generally localised to the lower lobes, in the peripheral and subpleural areas [12]. They have four characteristic features on CT: sharp, lobulated contours, calcification, and often central or in an irregular pattern within the nodule (seen in about 50% of cases) [13, 14], with multiple shapes and sizes varying from 0.5 to 15 cm [14] and slow growth, often over years, with no regression [12]. Cavitation is very rare [15]. Our patient's chest tomography showed multiple calcified nodules, air cysts in both lungs, and lymphadenopathy in the mediastinum. In the literature, there are limited studies on the mortality rate of interstitial lung disease in PSS. Although lung involvement is the third leading cause of death in patients with rheumatoid arthritis [16], the contribution of lung involvement to mortality in PSS is minimal [1, 7]. The 5-year survival rate for all PSS patients was reported as 84% [6]. Our patient was followed up without treatment for three years. There are only minimal lung symptoms related to lung infiltration.

In conclusion, pulmonary amyloidosis secondary to SS is an extremely rare and usually has a good survival even without treatment.
